# Claudin 5 Across the Vascular Landscape: From Blood–Tissue Barrier Regulation to Disease Mechanisms

**DOI:** 10.3390/cells14171346

**Published:** 2025-08-29

**Authors:** Mohamed S. Selim, Bayan R. Matani, Harry O. Henry-Ojo, S. Priya Narayanan, Payaningal R. Somanath

**Affiliations:** 1 Clinical and Experimental Therapeutics, College of Pharmacy, University of Georgia, Augusta, GA 30912, USA; 2 Department of Research, Veterans Affairs Augusta Health Care System, Augusta, GA 30912, USA; 3 Vascular Biology Center, Augusta University, Augusta, GA 30912, USA

**Keywords:** claudin 5, tight junction, endothelial-barrier, blood-tissue barrier, vascular permeability, organ injury

## Abstract

Claudin 5 (Cldn5) is a critical tight junction protein essential for maintaining paracellular barrier integrity across endothelial and epithelial cells in barrier-forming tissues, including the blood–brain barrier and blood–retinal barrier. Cldn5 plays a central role in regulating vascular permeability, immune responses, and tissue homeostasis. The complex distribution and organ-specific regulation of Cldn5 underscore its potential as a promising therapeutic target. This review comprehensively analyzes the role of Cldn5 in endothelial and epithelial barrier function, its regulation of vascular permeability, and the discrepancies in the literature regarding its expression, regulation, and function in both physiological and pathological conditions across multiple organ systems, including the retina, brain, lung, heart, gut, kidney, liver, skin, and peripheral nerves, while emphasizing its tissue-specific expression patterns. We discuss how both reduced and excessive expressions of Cldn5 can disrupt barrier integrity and contribute to the pathogenesis of ischemic retinopathies, neuroinflammation, cardiovascular injury, and other forms of barrier dysfunction. Furthermore, we explore the dual role of Cldn5 as both a biomarker and a therapeutic target, highlighting emerging strategies such as RNA silencing, pharmacological stabilizers, and transcriptional modulators in controlling barrier leakage in disease conditions.

## 1. Introduction

Blood-tissue barriers, formed by continuous, tightly connected layers of cells, include both endothelial barriers (lining blood and lymphatic vessels) and epithelial barriers (lining the outer surfaces of organs and body cavities) [1]. These barriers are maintained by specialized cell–cell junctions that preserve cellular integrity, polarity, and coordinated function [2,3,4]. Three major functional types of specialized cell junctions have been identified: tight junctions (TJs), anchoring/adherens junctions (AJs), and gap junctions (GJs) [5]. Among these, TJs consist of interconnected strands that connect the apical surfaces of adjacent cells and regulate paracellular permeability [6]. TJs were first identified in the 20th century using electron microscopy [7]. Despite their lipophilic and insoluble nature, TJs have been isolated, allowing for the characterization of their protein composition and their pivotal role in maintaining intracellular homeostasis. Beyond their role in maintaining barrier integrity, TJs also restrict the lateral movement of membrane components, including lipids and proteins, thereby contributing to the regulation of cell polarity [8]. The strength and integrity of TJs in both epithelial and endothelial cells can be experimentally evaluated using transepithelial electrical resistance (TEER), where higher TEER values indicate stronger barrier function [9].

TJs exhibit a complex molecular architecture formed by interactions between cytoplasmic adaptor proteins (e.g., zonula occludens) and transmembrane linker proteins, such as occludins and claudins. Initially, occludin was believed to be the primary transmembrane component of TJs. However, it was later shown that epithelial cells lacking occludin could still form intact TJ strands, suggesting that occludin is not solely responsible for TJ formation [10,11,12].

The discovery of the claudin (Cldn) family significantly advanced our understanding of TJ structure and function [13,14]. The term “claudins” is derived from the Latin word *claudere*, meaning “to close” [15]. In mammals, the Cldn family includes 27 integral membrane proteins localized to the apical side of epithelial cell–cell junctions, where they regulate the charge- and size-selective properties of the paracellular barrier [16]. The number of Cldn genes varies among species; for example, the teleost fish have an expanded Cldn repertoire to support their osmoregulatory needs [17]. Cldns are broadly classified into “classic” members (Cldns 1–10, –14, –15, –17, and –19) and “non-classic” members (Cldns –11, –12, –13, –16, –18, and –20–27) [18,19]. Classic claudins exhibit strong structural homology, whereas non-classic claudins are more structurally diverse [20].

Functionally, Cldns are categorized based on their permeability characteristics. “Tight” Cldns, including Cldns –1, –3, –4, –5, –6, –8, –12, –18, and –19, help restrict paracellular flux [21], while others, such as Cldn2 and Cldn15, are considered “leaky” or permeable [22,23]. Among these, Cldn5 is one of the most prominent “tight” claudins and is predominantly expressed in endothelial TJs, particularly within the blood–brain barrier (BBB) and the blood–retinal barrier (BRB) [24,25]. Cldn5 is also found in the endothelial and epithelial cells of the lung, kidney, liver, skin, and gut [26].

Cldn5 was initially discovered as a missing protein associated with the genetic disorder velo-cardio-facial syndrome, leading to its identification as the transmembrane protein deleted in velo-cardio-facial syndrome (TMVCF) [27]. Deletion of the *Cldn5* gene has also been implicated in two autosomal disorders, velo-cardio-facial syndrome and DiGeorge syndrome, both characterized by cleft palate, learning disabilities, distinctive facial features, and congenital heart defects [28].

Given the widespread tissue expression of Cldns, numerous studies have linked their dysregulated expression to various human diseases, including cancer, inflammation, metabolic disorders, and tissue dysfunction caused by compromised paracellular barriers [16,29,30,31]. This review will comprehensively explore the complex role of Cldn5 in regulating vascular permeability across various disease states and will highlight the ongoing controversy surrounding its expression and function in maintaining vascular health.

## 2. Physiology of Cldn5: Structure, Expression Regulation, Function, and Interactions

### 2.1. Structural Features and Protein Interactions

Cldn5 comprises four transmembrane domains, two extracellular loops (ECL1 and ECL2), and cytoplasmic N- and C-termini. The extracellular loops mediate homophilic and heterophilic interactions between adjacent cells, contributing to TJ strand formation and ion selectivity [26]. The C-terminal PDZ-binding motif anchors Cldn5 to intracellular scaffolding proteins, most notably the zonula occludens (ZO) family [26]. Among these, ZO-1 is a key adaptor that links Cldn5 to the actin cytoskeleton and coordinates the assembly of multiprotein junctional complexes. This interaction is essential not only for maintaining junctional architecture but also for transducing mechanical and signaling cues at the endothelial interface [19]. ZO-2 and ZO-3 may play supporting roles, but ZO-1 appears to be the primary partner in endothelial cells. Despite the functional importance of this interaction, contextual information about ZO-1 is often underrepresented in discussions of Cldn5, even though it is essential for TJ organization and stability [32].

### 2.2. Regulation of Cldn5 Expression

Cldn5 expression is tightly regulated at multiple levels, transcriptional, post-transcriptional, and post-translational, reflecting the sensitivity of vascular barriers to physiological and pathological stimuli [26]. At the transcriptional level, several key factors have been identified. FoxO1 acts as a transcriptional repressor of Cldn5 in endothelial cells, often in response to inflammatory or angiogenic signals [33,34,35,36]. Conversely, KLF4 [37] and Sp1 [38] promote Cldn5 transcription and are associated with barrier maintenance and endothelial differentiation. The Wnt/β-catenin pathway has also been shown to upregulate Cldn5 expression, particularly during central nervous system vascular development and in response to barrier injury, linking developmental and regenerative programs to barrier function [39].

Beyond transcription, Cldn5 is subject to post-transcriptional regulation. MicroRNAs such as miR-27a, miR-466, and miR-200 have been shown to downregulate Cldn5 translation, adding a layer of responsiveness to environmental cues [40,41]. Post-translationally, Cldn5 protein levels are influenced by internalization and degradation via lysosomal or proteasomal pathways [42]. Inflammatory mediators such as TNF-α and IL-1β promote Cldn5 endocytosis and degradation, contributing to barrier disruption during inflammation [19]. Growth factors, especially vascular endothelial growth factor (VEGF), reduce Cldn5 levels through both transcriptional downregulation and enhanced degradation, often via Src-family kinase signaling, further linking vascular permeability to angiogenic signaling [33,34,35]. Overall, Cldn5 expression is highly dynamic and integrates a broad range of inputs, from mechanical forces like shear stress to biochemical signals including cytokines, hypoxia, and metabolic cues.

### 2.3. Cell-Type and Organ-Specific Expression

**Figure 1:**
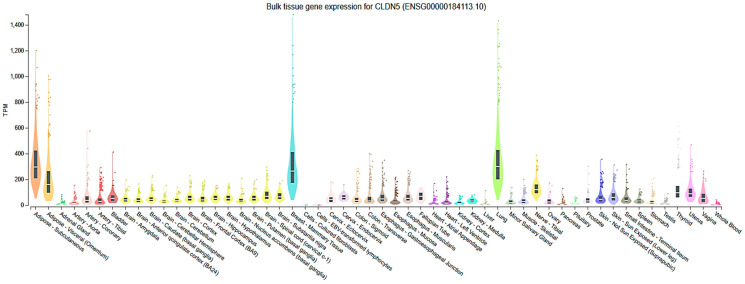
Genotype-Tissue Expression (GTEx) portal analysis (https://www.gtexportal.org/home/ accessed on 18 June 2025) of Cldn5 in various human tissues shows predominant Cldn5 mRNA expression in tissues such as the subcutaneous and visceral adipose tissues, blood vessels, brain, breast, bladder, intestine, lungs, nerves, skin, and uterus among other tissues.

Cldn5 is expressed almost exclusively in endothelial cells and is a hallmark of vascular identity, particularly in the central nervous system [19], retina [43,44,45], and the lungs [33]. Within the brain, it is specifically enriched in brain microvascular endothelial cells (BMECs) that form the BBB [19]. Outside the CNS, Cldn5 is also found in endothelial cells of the lung, heart, kidney, liver, and skin, although expression levels and functional roles vary by tissue and vascular bed [26] (Figure 1). Cldn5 is weakly expressed in epithelial cells, along with other Cldns such as Cldn1, Cldn3, or Cldn4, which are common in epithelial TJs [26]. Higher endothelial-specific expression has implications for both its physiological function and its dysregulation in disease states.

## 3. Cldn5 in Endothelial-Barrier Regulation

The Src and Akt signaling pathways play pivotal roles in regulating endothelial barrier integrity and vascular permeability [46,47], subsequently revealing the importance of Cldn5 in this process [34,35,36,48]. A growing body of cellular research has highlighted the dynamic regulation of Cldn5 in endothelial cells under inflammatory, infectious, and tumorigenic conditions. In human microvascular endothelial cells (HMVECs) challenged with lipopolysaccharide (LPS), cisatracurium preserved junctional integrity by preventing Cldn5 degradation and concurrently reducing MMP3 expression [49]. The Akt–FoxO axis has emerged as a key regulator of Cldn5 expression and TJ integrity [35,36]. In LPS-induced lung injury, activation of FoxO1/3 transcription factors suppresses endothelial Akt signaling, a pathway increasingly recognized not only for its role in cell survival but also for maintaining TJ integrity [33]. This signaling shift led to marked downregulation of Cldn5, compromised junctional structure, and increased paracellular permeability in pulmonary microvascular endothelial cells.

Similarly, endothelial-specific loss of Akt1 promotes nuclear accumulation of β-catenin, which represses Cldn5 transcription, disrupts barrier function, and facilitates prostate cancer cell transmigration, lung metastasis, and diabetes-enhanced carcinogenesis [35,36,48,50]. These findings demonstrate the central role of Akt1 in preserving Cldn5 expression and maintaining endothelial homeostasis, thereby preventing FoxO– and β–catenin–mediated barrier destabilization. Sustained Akt activation, alongside Src signaling, is essential for maintaining endothelial barrier integrity by regulating TJ proteins, particularly Cldn5 [34]. While Src mediates acute permeability changes, long-term barrier stabilization depends heavily on Akt–FoxO–mediated Cldn5 preservation.

New insights reveal that the chemokine CCL2 induces caveolin-dependent endocytosis of Cldn5 and occludin in brain endothelial cells, resulting in decreased barrier resistance and pointing to endocytosis as a critical mechanism of junctional remodeling [51]. Intriguingly, in an airway-on-a-chip model, SARS-CoV-2 exposure suppressed Cldn5 expression in the vascular respiratory endothelium, leading to barrier disruption. This effect was reversed by Cldn5 overexpression or fluvastatin treatment [52]. In contrast, Cryptococcus neoformans infection paradoxically upregulated Cldn5 expression in brain endothelial cells, a response reversed by candesartan and triciribine treatment [53].

**Figure 2:**
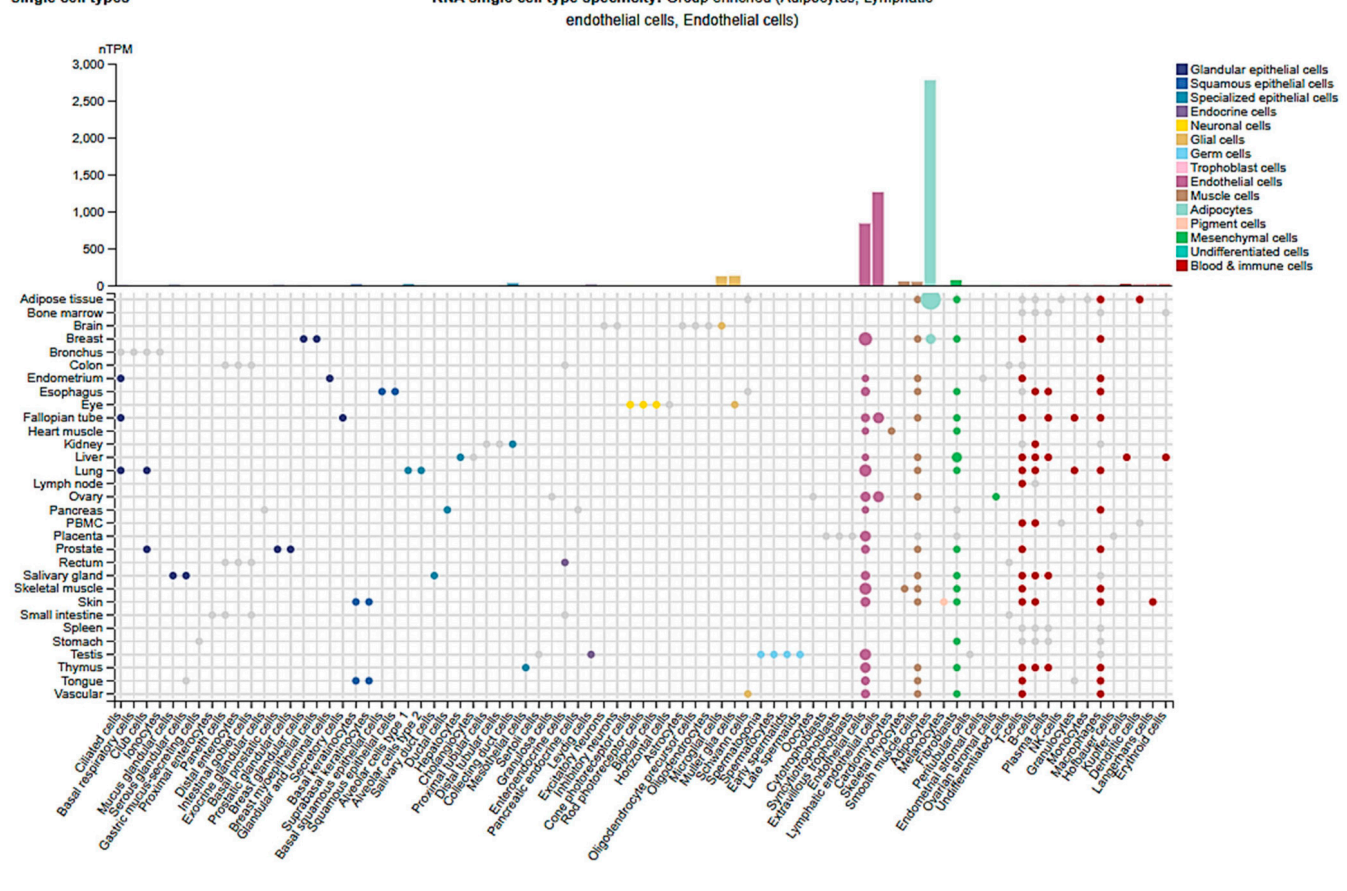
Single-cell transcriptomic analysis of Cldn5 expression across human cell types in various tissues. Data retrieved from the Protein Atlas database (https://www.proteinatlas.org/ accessed on 18 June 2025) reveals cell-type-specific Cldn5 mRNA expression patterns across diverse human tissues. High Cldn5 expression was observed in endothelial cells across multiple organs, with detectable levels also present in select epithelial, smooth muscle, and immune cell populations. This distribution highlights the preferential expression of Cldn5 in vascular-associated cells, consistent with its role in maintaining barrier integrity.

In an alternate pathway, JAM-A promotes endothelial barrier integrity by activating C/EBPα, a transcription factor that directly upregulates Cldn5. JAM-A–deficient cells and mice exhibited significantly reduced Cldn5 levels, correlating with increased vascular permeability [54]. Additionally, VE-cadherin clustering enhances Cldn5 expression by inhibiting nuclear localization of β-catenin and FoxO1, revealing an epigenetic mechanism in which adherens junctions influence TJ gene activation and vascular stability [36,55]. Together, these studies underscore the complex, context-dependent regulation of Cldn5 and its critical role in maintaining and restoring endothelial homeostasis. Single-cell analysis data from the Protein Atlas database confirmed the predominant expression of Cldn5 in lymphatic and vascular endothelial cells, among others (Figure 2).

While Cldn5 is primarily known for its classical role in regulating paracellular permeability and maintaining endothelial barrier integrity, accumulating evidence suggests it may also contribute to non-canonical functions that go beyond passive barrier formation. These include roles in endothelial cell signaling, cytoskeletal organization, and cellular responses to stress or inflammation [19]. For instance, Cldn5 loss has been linked to changes in endothelial gene expression profiles, including inflammatory and angiogenic pathways, suggesting it may indirectly influence cell behavior through transcriptional or signaling feedback loops [56]. Additionally, its interaction with adaptor proteins like ZO-1 may facilitate communication with intracellular signaling hubs that modulate cell proliferation or polarity [57]. Although these downstream effects are not yet fully understood, they point to a broader regulatory capacity for Cldn5 beyond simple barrier function, particularly in disease contexts where its expression is dysregulated.

## 4. Cldn5 and BBB Permeability Modulation in Health and Disease

**Figure 3:**
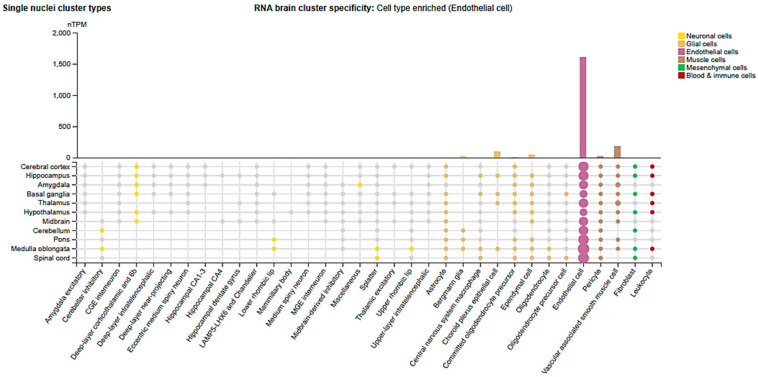
RNA expression of Cldn5 across human brain regions and cell clusters. Data from the Protein Atlas brain RNA cluster analysis (https://www.proteinatlas.org/ accessed on 18 June 2025) shows that Cldn5 expression is highly enriched in vascular and endothelial cell clusters throughout various brain regions, including the cortex, hippocampus, cerebellum, and basal ganglia. This pattern underscores the brain region–independent vascular specificity of Cldn5, consistent with its established role in maintaining BBB integrity.

Cldn5 is highly enriched in brain microvascular endothelial cells (BMECs) (Figure 3) and is fundamental for maintaining BBB integrity and regulating brain neuro-trafficking [26]. Genetic knockout studies revealed that loss of Cldn5 results in a size-selective loosening of the BBB, allowing tracers between 0.5–1 kDa to penetrate the brain without overt vascular disruption [58]. This subtle BBB compromise has also been identified as an early event preceding cognitive impairment in aging human brains [59].

The essential role of Cldn5 in the brain is evolutionarily conserved. In zebrafish, which harbor two paralogs of Cldn5 (cldn5a and cldn5b). The loss of Cldn5a, which is the functional ortholog most similar to mammalian Cldn5 and expressed in BBB ECs, caused severe brain edema and herniation, despite preserved vascular morphology, highlighting isoform-specific functions [60]. In human disease, Cldn5 downregulation is a hallmark of BBB disruption. For example, its reduced expression in amyloid-laden microvessels of patients with cerebral amyloid angiopathy has been linked to cerebral hemorrhage [61]. Likewise, diminished Cldn5 levels are reported in epilepsy, traumatic brain injury (TBI), and multiple sclerosis (MS), where its loss correlates with increased barrier permeability and neuroinflammation [62,63].

Importantly, restoring or stabilizing Cldn5 expression confers neuroprotection. In preclinical epilepsy models, microvascular Cldn5 stabilization reduced seizure susceptibility and neuroinflammation [64]. Similarly, Cldn5 overexpression in brain endothelial cells (hCMEC/D3) enhanced proliferation, migration, and adhesion, strengthening the barrier and limiting lung cancer metastasis to the brain [65].

Extracellular vesicles (EVs), nanosized particles that facilitate intercellular communication, have emerged as key players in Cldn5 dynamics [66]. In MS, circulating endothelial-derived EVs transfer Cldn5 to infiltrating leukocytes, leading to the appearance of Cldn5^+^ immune cells in the CNS [67]. This suggests a novel immune trafficking function for Cldn5. In the EAE mouse model of MS, CD4^+^ and CD8^+^ T cells, B cells, monocytes, and neutrophils were found to ectopically express Cldn5 in both blood and CNS [68]. Notably, Cldn5^+^ T cells showed greater activation, indicating EV-mediated endothelial-to-leukocyte transfer facilitates immune infiltration across the BBB. In MS patients, fingolimod treatment reduced circulating TJ proteins, including Cldn5, which was associated with fewer new brain lesions, suggesting stabilization of the BBB via inhibition of TJ protein shedding and immune cell trafficking [69]. In contrast, EVs from preeclamptic women or hypoxic placental explants were reported to disrupt BBB integrity by depleting Cldn5 in human and mouse brain endothelial cells, leading to increased permeability, particularly in the posterior cortex. These EVs were enriched in VEGF, and their disruptive effect was reversed by sonication, confirming the role of EV cargo in Cldn5 downregulation [70]. This suggests that, while EVs can deliver Cldn5 in immune-driven neuroinflammation, they can also deplete or redistribute Cldn5 in systemic disorders, such as preeclampsia, suggesting context-specific, bidirectional regulation of endothelial barrier integrity via EVs.

Metabolic stress also disrupts Cldn5 regulation. Exposure of BMECs to high glucose (HG) and advanced glycation end-products (AGE) triggered Cldn5 and occludin shedding via EVs, exacerbating barrier breakdown [71]. In contrast, mesenchymal stem cell–derived EVs protected the BBB in acute ischemic stroke by inhibiting Caveolin-1–mediated endocytosis of Cldn5 and ZO-1, preserving their membrane localization and improving neurological outcomes [72]. Likewise, AY9944, an experimental Caveolin-1 inhibitor, reduced Caveolin-1 expression and increased Cldn5 levels in brain endothelium, resulting in reduced edema and infarct size in ischemic brain injury [73]. Mechanistically, membrane-bound Cldn5 is vulnerable to redistribution under hypoxia or ischemia, accumulating in the cytosol and weakening TJs. Autophagy mitigates this by preserving membrane-localized Cldn5 and preventing degradation [74]. Additionally, inflammatory cytokines such as IL-1β suppress Cldn5 by inhibiting the PI3K/AKT2/FOXO1 pathway, an effect reversed by insulin receptor activation [63].

Neuro-glial interactions also modulate Cldn5. Coculturing porcine brain endothelial cells with rat glial cells markedly upregulated Cldn5 expression, highlighting glial influence on TJ enhancement [75]. Echoing these results, in a triple co-culture BBB model, co-culturing brain ECs with astrocytoma U251 and neuroblastoma SH-SY5Y cells significantly increased Cldn5 and VE-cadherin expression via GDNF secretion and activation of PI3K/AKT/FOXO1 and MAPK/ERK pathways [76].

Human studies on Cldn5 show conflicting findings. Elevated serum Cldn5 levels are reported in schizophrenia, bipolar disorder, depression, and OCD, whereas increased occludin is noted in ADHD and autism [77,78,79], suggesting shared BBB dysfunction across psychiatric conditions. However, this interpretation is far from settled: one case–control study reported lower serum Cldn5 levels in schizophrenia compared to healthy controls [78], pointing to temporal or population-specific variability.

Growing evidence implicates the dysregulation of Cldn5 in Alzheimer’s disease (AD), highlighting its emerging role in maintaining neurovascular integrity beyond classical amyloid and tau pathology. Post-mortem analysis of 40 human AD brains has revealed a selective downregulation of Cldn5 expression, particularly in the neocortex. This reduction was associated with elevated amyloid-β_40_ levels and synaptic loss, even in the absence of significant tau buildup [80]. Intriguingly, in a cohort study, plasma Cldn5 levels were elevated in patients with mild cognitive impairment and AD but declined with age in these patients. This suggests an early compensatory release of Cldn5 during initial BBB disruption, followed by depletion as barrier breakdown progresses [56,81]. Epigenetic studies further confirmed the relevance of Cldn5. DNA methylation of Cldn5 was strongly linked to accelerated cognitive decline, even in individuals with low amyloid/tau burden, implying that vascular and barrier dysfunction may precede traditional AD pathology [82]. Similarly, in aged and AD mouse models, Cldn5 was downregulated in diseased animals, while intravenous (i.v.) administration of recombinant, full-length Cldn5 improved memory function in mice. Furthermore, knocking out Cldn5 impaired cognition and long-term memory even in healthy mice, highlighting a functional neurovascular role of Cldn5 in cognitive performance [83]. In both human and mouse AD models, exposure to amyloid-β (Aβ42 oligomers) directly reduces endothelial Cldn5, impairing the barrier integrity and contributing to progressive neurodegeneration [84]. The phenomenon of circulating Cldn5 in the blood, often in EVs, is increasingly reported in various diseases and provides a novel window into blood-tissue barrier status. For instance, EV-containing Cldn5 were detected in blood under neuroinflammatory conditions [70], venous blood levels were significantly elevated in patients with OCD compared to healthy controls [85], and serum levels were found to be decreased in patients with schizophrenia [78].

Adding a genetic dimension, the functional Cldn5 variant rs885985, which reduces expression, was linked to stress-induced depression via increased BBB leakiness to IL-6, particularly in carriers of the IL6 variant rs1800795, indicating a gene–environment interaction that links endothelial dysfunction to depressive symptoms [86]. In HIV-associated neurocognitive disorders, a viropathological mechanism disrupts Cldn5. HIV-1 Tat protein downregulates Cldn5 mRNA and protein in brain ECs, impairing TJ localization and BBB integrity [87,88]. Autopsied brain microvessels from patients with HIV showed reduced or fragmented Cldn5 immunoreactivity, confirming BBB compromise [89].

Therapeutically, targeting Cldn5 holds both promise and risk. A low IV dose of anti-Cldn5 monoclonal antibody improved cerebrospinal fluid tracer penetration in non-human primates. However, higher doses caused convulsions and vascular injury, demonstrating a narrow therapeutic window. Furthermore, a similarly narrow margin was evident in rodents, where sustained suppression of Cldn5 was lethal in weeks, whereas transient siRNA knockdown remained tolerable [90,91]. Similarly, M01, a Cldn5 inhibitor, transiently opened the blood–spinal cord barrier by inducing Cldn5 internalization and downregulation, thereby reducing neuroinflammation and vasogenic edema post–spinal cord injury [92].

In summary, Cldn5 is a pivotal gatekeeper of the BBB [26]. Its downregulation is implicated in a range of neuropathologies, while controlled upregulation can restore barrier integrity and curb disease progression. However, its role is highly context-dependent: Cldn5 may paradoxically facilitate immune cell infiltration via EV-mediated transfer. Elevated serum Cldn5 might indicate disrupted localization, not protective expression. Therapeutic strategies must therefore consider disease-specific context to balance the benefits and risks in modulating Cldn5.

## 5. Cldn5 in Retinal Vascular Pathophysiology

### 5.1. Diabetic Retinopathy

Diabetic retinopathy (DR), one of the most prevalent microvascular complications linked to diabetes, is the leading cause of vision loss among adults in the United States [93]. DR progresses from non-proliferative (NPDR) to proliferative (PDR) stages, with both forms characterized by increased paracellular permeability. However, PDR is particularly marked by extensive pathological angiogenesis [94]. Over the years, multiple therapeutic strategies have been employed to combat DR, including laser photocoagulation, anti-VEGF therapy, intravitreal corticosteroid injections, and vitreoretinal surgeries [95,96]. While effective to varying degrees, these treatments are often costly and carry significant side effects, whereas surgical approaches are typically reserved for advanced stages with severe vision loss [97].

A critical factor underlying DR pathogenesis is the dysfunction of the BRB, which is essential for maintaining retinal homeostasis [5,44,98]. The BRB consists of two primary components: the outer BRB (oBRB), comprising the choroid, Bruch’s membrane (BM), and the retinal pigment epithelium (RPE), and the inner BRB (iBRB), formed by glial cells, pericytes, and endothelial cells lining the retinal vasculature [44,98]. The iBRB plays a central role in maintaining the integrity of the retinal environment and is a major site of disruption in DR [44,99]. The iBRB function is heavily reliant on the integrity of TJs, which regulate solute diffusion and prevent toxicants and inflammatory cells from infiltrating the retina [100]. Cldn5 is one of the most abundantly expressed TJ proteins in the iBRB [24]. Its expression is regulated by circadian rhythms, with lower expression levels observed in retinal microvasculature during the evening compared to the morning in mice [101]. In the oBRB, transient upregulation of Cldn5 occurs in the RPE during early chick embryonic development, coinciding with increasing membrane selectivity and decreasing permeability [102]. While chronic suppression of Cldn5 in mice maintained on a cholesterol-enriched diet induced marked RPE atrophy, sustained region-specific knockdown of Cldn5 in the macula of nonhuman primates resulted in RPE degeneration [101].

Given its high expression in the BRB, Cldn5 plays a significant role in regulating retinal vascular permeability. Both upregulation and downregulation of Cldn5 have been shown to impact vascular integrity. In human retinal endothelial cells (HRECs) exposed to HG, Cldn5 expression is reduced compared to normal glycemic conditions, resulting in increased paracellular permeability [45,103]. Similarly, temporal downregulation of occludin and Cldn5 has been documented in STZ-induced diabetic rat retina and VEGF-treated bovine retinal endothelial cells (BRECs), contributing to increased capillary leakage [104]. Extending this pattern, osteopontin, a phosphoglycoprotein elevated during retinal ischemia and inflammation [105], downregulated both Cldn5 and ZO-1 expression, disrupting endothelial barrier function and increasing permeability in STZ-induced diabetic mice [106]. Elevated matrix metalloproteinases (MMP-2 and MMP-9) in HG-treated BRECs mediate proteolytic degradation of TJ complexes, including Cldn5, resulting in decreased TEER in vitro and increased retinal capillary permeability in diabetic rats in vivo [107].

Adequate oxygenation is essential for the expression of various factors, including growth factors, cytokines, and enzymes [108]. Hypoxic conditions can compromise barrier function, accelerating cerebrovascular ischemic diseases, including DR [109]. In this context, Cldn5 expression is markedly reduced under hypoxia in both brain-derived endothelial (bEND.3) and retinal cells, leading to decreased TEER and increased retinal permeability in vitro and in vivo [109]. Conversely, excessive Cldn5 expression may also impair barrier function. In HRECs treated with HG and AGE, Cldn5 expression and junctional accumulation increased, paradoxically leading to impaired barrier resistance [45].

Emerging studies highlight the role of EVs in mediating vascular dysfunction in DR. In the vitreous of DR patients, Cldn5^+^ EVs were significantly elevated compared to non-diabetic controls [110]. When THP-1 macrophages were treated with these EVs, expression of pro-inflammatory cytokines such as TNFα and IL-1β was upregulated, suggesting a role in perpetuating retinal inflammation [110]. Proteomic analysis of these EVs revealed a complex cargo likely of endothelial origin, reflecting ongoing vascular inflammation and BRB disruption [110]. Additionally, exosomes, a subset of EVs (30–150 nm), have been implicated in promoting microvascular damage in STZ-induced diabetic mouse models [111]. In agreement, HG and AGE drive BBB disruption in diabetes through increased Cldn5 and occludin expression, highlighting BBB protection as a potential therapeutic strategy to prevent diabetes-associated cognitive decline [112]. Collectively, these findings substantiate the complex and context-dependent role of Cldn5 in the regulation of BRB integrity during diabetic retinopathy, where both loss and excess of this TJ protein can disrupt vascular homeostasis and contribute to disease progression.

### 5.2. Diabetic Macular Edema

The second most prevalent ocular complication in diabetes is diabetic macular edema (DME), which arises as a primary consequence of diabetic retinopathy (DR) [113]. The macula, the central region of the retina responsible for high-acuity vision, becomes particularly vulnerable in DME due to chronic vascular leakage and fluid accumulation stemming from untreated or progressive DR. This fluid buildup results in neural cell damage and progressive, often irreversible, loss of central vision [114].

VEGF has emerged as a pivotal driver of DME pathophysiology. VEGF is a potent vascular permeability factor that promotes inflammation, enhances fluid accumulation, and disrupts the TJs that preserve the BRB integrity [115,116]. A study has demonstrated that VEGF overexpression upregulates several pro-inflammatory cytokines, including tumor necrosis factor α (TNFα), interleukin-6 (IL-6), and monocyte chemoattractant protein-1 (MCP-1), and contributes to the destabilization of Cldn5, a critical TJ protein in retinal endothelial cells [117].

In diabetic mouse models, VEGF-induced inflammation led to decreased Cldn5 expression and reduced TEER in brain-derived endothelial cells (bEND.3). Notably, these effects were not fully reversed by anti-VEGF therapy alone, indicating the presence of VEGF-independent mechanisms or compensatory pathways contributing to sustained barrier dysfunction [117]. One such pathway involves the activation of Rho-associated coiled-coil–containing protein kinase-1 (ROCK1), a downstream effector of VEGF signaling implicated in cytoskeletal remodeling and endothelial permeability. Inhibition of ROCK1 was shown to suppress VEGF-induced inflammatory cytokines, restore Cldn5 expression and localization, and enhance Cldn5 transcription in both Kimba diabetic mice and HRECs [117]. This intervention ultimately preserved the integrity of the retinal vascular barrier. Taken together, these findings highlight a crucial VEGF–ROCK1–Cldn5 regulatory axis in the pathogenesis of DME. Targeting ROCK1, in conjunction with or as an alternative to anti-VEGF therapy, may provide a promising therapeutic strategy, particularly in cases where VEGF inhibition alone is insufficient to restore retinal barrier function.

### 5.3. Retinopathy of Prematurity

Retinopathy of prematurity (ROP) is a proliferative retinal disease that affects premature infants and remains one of the leading causes of childhood blindness since it was first described in the 1940s [118]. ROP develops in two distinct phases. The first phase involves the suppression of normal retinal vascular development and regression of pre-existing vessels, while the second phase is marked by hypoxia-induced pathological neovascularization as the metabolically active retina demands revascularization [119]. The oxygen-induced retinopathy (OIR) mouse model is widely used to study ROP pathogenesis. In this model, Cldn5 expression is significantly upregulated at both gene and protein levels [120]. However, this increase is not functionally protective; rather, it coincides with aberrant localization of Cldn5 to cytosolic or non-junctional regions of the plasma membrane, which contributes to increased vascular permeability and impaired barrier function [121]. Consistent with these findings, our laboratory has also demonstrated pathological upregulation of Cldn5 in the OIR model, where elevated levels contributed to retinal vascular barrier dysfunction [43,122]. Pharmacological intervention with Triciribine, a small-molecule inhibitor with known anti-angiogenic properties, was shown to reverse the pathological effects of Cldn5 overexpression in the OIR model [43]. Treatment resulted in a marked reduction in neovascular tuft formation and vascular leakage, highlighting the potential therapeutic benefit of targeting Cldn5 dysregulation. These results agree with the effect of HG and AGE on HRECs in vitro [45].

Further evidence supports the pro-angiogenic role of Cldn5 during both developmental and pathological retinal vascularization. In HRECs, antibody-mediated inhibition of Cldn5 significantly suppressed angiogenic sprouting that was otherwise stimulated by Wnt ligands (Wnt3a, Wnt7a) or VEGF [123]. Similarly, developmental angiogenesis in neonatal mouse retinas was attenuated [123]. In vivo, intravitreal injection of Cldn5 siRNA effectively suppressed pathological neovascularization in the OIR model, reducing neovascular changes at postnatal day 14 (P14) and continuing through P17 [123]. These findings collectively indicate that, beyond its canonical role in maintaining the BRB, Cldn5 also facilitates angiogenesis under both physiological and pathological conditions. Therefore, suppressing aberrant Cldn5 expression or function may serve as a viable strategy to mitigate pathological neovascularization in ischemic retinopathies such as ROP.

### 5.4. Stressors and Cytokine-Mediated Disruption of Cldn5 and Retinal Barrier Integrity

Retinal Cldn5 is highly susceptible to disruption by various exogenous and endogenous stressors. These include environmental particulates, pro-inflammatory cytokines, and intracellular stress pathways, all of which have been shown to impair Cldn5 expression or localization, leading to increased retinal permeability and barrier dysfunction [124]. Environmental exposure to titanium dioxide (TiO_2_) nanoparticles, commonly found in household products such as toothpaste, food additives, and cosmetics, has been demonstrated to suppress Cldn5 expression in HRECs. This suppression compromises barrier integrity, as evidenced by reduced TEER values. Moreover, TiO_2_ exposure directly activates ADAM17, a disintegrin and metalloprotease that accelerates Cldn5 degradation, further compounding the breakdown of retinal endothelial junctions [125].

Inflammatory cytokines also play a central role in disrupting Cldn5. Transforming growth factor-β1 (TGF-β1), a multifunctional cytokine involved in immune modulation, wound healing, and fibrosis [126], has been shown to increase paracellular permeability in a dose-dependent manner in bovine retinal endothelial cells (BRECs) and human cerebral endothelial cells (hCMEC/D3). Mechanistically, TGF-β1 promotes tyrosine phosphorylation of Cldn5 and VE-cadherin, leading to their downregulation and loss of junctional integrity [127]. Similarly, pro-inflammatory cytokines such as tumor necrosis factor-α (TNF-α) and interleukin-1β (IL-1β) elevate paracellular permeability in both bovine retinal vascular endothelial cells (BRVECs) and mouse retinas by reducing the mRNA and protein expression of Cldn5 and ZO-1. Among the two, TNF-α exerts a more potent barrier-disrupting effect [128]. Notably, dexamethasone effectively counteracts these inflammatory insults by inhibiting TNF-α–induced nuclear factor kappa B (NF-κB) activation, thereby preserving Cldn5 expression and BRB function [124]. Our laboratory has demonstrated that LPS reduces Cldn5 expression in HRECs in vitro, as well as in a mouse model of LPS-induced uveitis-associated retinal injury [45,129].

Cellular stress responses also significantly impact Cldn5 integrity. Treatment of HRECs with endoplasmic reticulum (ER) stress inducers, thapsigargin (Tg) and tunicamycin (Tm), results in a substantial decrease in both Cldn5 expression and TEER, which increases paracellular permeability [130]. Importantly, pharmacologic inhibition of p38 mitogen-activated protein kinase (MAPK) and NF-κB signaling pathways mitigated Tg-induced Cldn5 suppression and restored barrier resistance, suggesting that these stress pathways mediate inflammation-driven loss of Cldn5 and BRB integrity [130]. Together, these findings illustrate that Cldn5 is a convergent target of diverse pathological stimuli. Its disruption represents a unifying feature of BRB breakdown in response to environmental toxins, inflammatory cytokines, and intracellular stress. These insights support the consideration of Cldn5 as a key molecular determinant of retinal vascular integrity and a potential therapeutic target for preserving BRB function under stress conditions.

**Figure 4:**
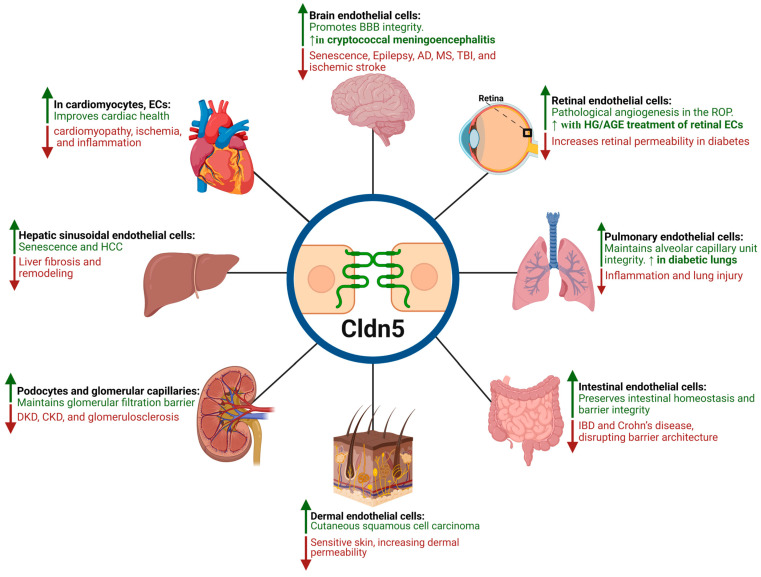
Schematic overview of Cldn5 regulation across tissues under physiological and pathological conditions. This illustration summarizes the modulation of Cldn5 expression in diverse tissues in response to varying environmental, inflammatory, and disease-related stimuli. Differential expression patterns are linked to functional consequences on barrier integrity, tissue homeostasis, and the progression of pathological processes such as inflammation, fibrosis, and vascular leakage. ↑ Upregulation; ↓ Downregulation.

Figure 4 illustrates a schematic representation of how Cldn5 expression is modulated across various tissues under different conditions, highlighting its implications in both physiological and pathological contexts. A more detailed summary of Cldn5 expression, functions, and regulation across these tissues in health and disease is provided in Table 1.

## 6. Cldn5 in Peripheral Nerves and Cranial Nerve Barriers

Studies on inflammatory neuropathies highlight how the loss of Cldn5 facilitates immune-mediated nerve inflammation. Guillain–Barré syndrome (GBS) and chronic inflammatory demyelinating polyneuropathy (CIDP) are diseases characterized by blood–nerve barrier (BNB) disruption, allowing immune cells to infiltrate and attack peripheral nerve myelin [131]. Immunohistochemical analysis of sural nerve biopsies from CIDP patients revealed significantly reduced Cldn5 expression at endoneurial endothelial junctions, even in regions lacking overt edema, suggesting that immune processes selectively downregulate Cldn5, potentially increasing barrier permeability to leukocytes and pathogenic antibodies [131]. Supporting this concept, in vitro studies demonstrated that hydrocortisone, a glucocorticoid commonly used in neuropathy treatment, markedly upregulates Cldn5 in cultured endoneurial endothelial cells, leading to tighter BNB integrity and reduced permeability [132]. This restorative effect likely contributes to the clinical efficacy of corticosteroids in managing immune-mediated neuropathies.

Similarly, glial cell line-derived neurotrophic factor (GDNF) released by pericytes has been shown to induce Cldn5 expression in both the BBB and blood–nerve barrier (BNB), highlighting the role of perivascular support cells in maintaining junctional integrity within nerve barriers [133]. Taken together, evidence from peripheral nerve disorders reinforces the critical role of Cldn5 as a key regulator of immune cell infiltration across the BNB and supports its potential as a therapeutic target in inflammatory neuropathies.

## 7. Cldn5 in Cardiovascular Diseases and Organ Injuries

### 7.1. Heart Failure and Cardiomyopathy

Cldn5 is expressed along the lateral membranes of cardiomyocytes, particularly at the junctions with the extracellular matrix, as well as throughout the endothelial lining of cardiovascular structures [135]. Beyond its classical role in TJs, Cldn5 was found to co-localize with mitochondria in human AC16 cardiomyocytes, implicating it in intracellular organelle regulation [134]. Knockdown of Cldn5 led to increased mitochondrial fission and heightened susceptibility to ischemic and hypoxic stress, suggesting a protective role in mitochondrial integrity [134]. Supporting this, Cldn5 was significantly downregulated at the cardiomyocyte lateral membrane in a dystrophin–utrophin double knockout (*Dmdmdx;Utrn*^−/−^ dko) mouse model that recapitulates features of cardiomyocyte degeneration and myocarditis, implicating Cldn5 loss in cardiomyopathy pathogenesis [135]. Notably, adenovirus-mediated Cldn5 overexpression (*rAAV6-Cldn5* dko) in this model for 4 weeks inhibited hallmark features of cardiomyopathy and improved histological indicators of cardiac injury [136]. Similarly, Cldn5 expression was markedly reduced in a mouse model of acute myocardial ischemia–reperfusion (IR) injury and in HL-1 cardiomyocytes exposed to hypoxia–reoxygenation (HR) conditions [134].

Cardiac-specific Cldn5 overexpression in mice led to attenuation of oxidative stress, inflammation, apoptosis, and cardiac dysfunction, demonstrating its cardioprotective function in vivo [137]. Estrogen has been shown to regulate Cldn5 expression in the cardiovascular endothelium. Treatment of microvascular myocardial endothelial cells, expressing both ERα and Erβ, with 17β-estradiol resulted in enhanced TEER and a marked upregulation of Cldn5 [166]. This significance is not limited to preclinical models. In atrial fibrillation (AF) patients, Cldn5 expression was significantly reduced in the left atrial appendage, and accompanying proteomic analysis revealed dysregulation of proteins associated with dilated cardiomyopathy [138]. Consistently, Cldn5 levels were downregulated in human heart tissues from patients with ventricular dysfunction caused by both ischemic and non-ischemic cardiomyopathies, compared to healthy controls [139]. Likewise, substantial loss of Cldn5 was observed in both cardiomyocytes and endothelial cells in failing human heart tissues relative to non-failing hearts [140]. Taken together, these findings establish Cldn5 as a multifunctional protein involved in maintaining cardiomyocyte structure, mitochondrial homeostasis, and endothelial integrity. Its downregulation is consistently associated with structural and functional abnormalities in various cardiomyopathies, highlighting its potential as both a biomarker and therapeutic target in cardiovascular disease.

### 7.2. Lung-Air Barrier Dysfunction

The pulmonary endothelial microvasculature abundantly expresses Cldn5 [141]. In addition to its vascular localization, Cldn5 is also present in the mature alveolar epithelium, where it contributes to epithelial barrier function [167]. Together, the pulmonary microvascular endothelium and alveolar epithelium reinforce the alveolar air–liquid interface, forming a dual-layered barrier essential for gas exchange and fluid homeostasis. Functionally, Cldn5 enhances barrier integrity and limits vascular leakage, highlighting its protective role in lung homeostasis [168].

Environmental and infectious stressors dynamically regulate Cldn5 expression. For example, exposure to hydrogen sulfide, a noxious environmental gas, induced acute lung injury in a murine model by suppressing Cldn5 expression. Treatment with dexamethasone attenuated this injury through activation of the PI3K/AKT/FoxO1 signaling pathway, indicating a mechanistic axis for therapeutic intervention [142]. Similarly, HIV-1 infection was shown to downregulate Cldn5 in pulmonary endothelial cells, contributing to vascular injury and interstitial pneumonitis [143]. In addition, systemic inflammation from bacterial lung infections was found to disrupt the BBB by downregulating Cldn5 in response to pro-inflammatory cytokine signaling, rather than through direct bacterial invasion, emphasizing a lung–brain inflammatory axis [144].

Beyond barrier function, Cldn5 may also act as a tumor suppressor in lung cancer. Expression of Cldn5 was significantly reduced in lung squamous cell carcinoma (SCC) tissues compared to normal lung tissues [145]. Reintroduction of Cldn5 into RERF-LC-AI human lung SCC cells led to reduced proliferation, mediated via AKT pathway inhibition, underscoring its oncostatic potential [145].

Interestingly, Cldn5 appears to have a cell-type specific role in the lung. While its expression in endothelial cells strengthens barrier function, overexpression in alveolar epithelial cells paradoxically disrupted barrier integrity, indicating context-dependent effects [147]. Recent studies in diabetic models further support this dichotomy. In AGE-treated lung epithelial and endothelial cells, Cldn5 overexpression impaired barrier function and significantly decreased TEER, suggesting organ- and disease-specific vulnerabilities [148,149]. Furthermore, plasma Cldn5 levels were found to be reduced in patients with stable asthma but significantly increased during exacerbations, hinting at systemic endothelial activation or microvascular leakage in severe airway inflammation [169]. A similar pattern was observed in an ovalbumin-induced allergic asthma mouse model, where Cldn5 transcript and protein expression were upregulated in lung tissue. This elevation was reversed by corticosteroid treatment, indicating that anti-inflammatory interventions can modulate Cldn5 expression and release [169].

Preclinical studies have identified promising therapeutic avenues. For instance, simvastatin enhanced pulmonary endothelial barrier integrity in a murine model of acute lung injury by upregulating Cldn5 via a VE-cadherin–dependent mechanism involving FoxO1 and β-catenin, ultimately reducing paracellular permeability to small molecules [146]. Mice treated with triciribine to inhibit the hyperactive Akt pathway in ARDS lungs protected the lungs from LPS-induced lung injury and inflammation via FoxO inhibition, MMP3 suppression, and Cldn5 preservation [170]. Collectively, these findings corroborate the critical role of Cldn5 in preserving lung vascular integrity. Its downregulation is implicated in the pathogenesis of numerous acute and chronic pulmonary disorders, including infection, inflammation, toxic exposure, and malignancy, and its excessive expression is correlated with lung injury in diabetic mice [171]. Therefore, therapeutic strategies aimed at restoring or modulating Cldn5 expression may offer significant clinical benefit in lung diseases.

### 7.3. Liver Physiology and Pathology

While Cldn5 function is well characterized in the BBB, BRB, and the lungs, accumulating evidence suggests it may also play a regulatory role in liver physiology and pathology [150]. Cldn5 is constitutively expressed in sinusoidal endothelial cells (SECs), as well as in the arterial and portal veins of healthy liver tissue, with a limited but increased expression in hepatocytes with pathology [172]. Notably, a reduction in sinusoidal Cldn5 expression correlates with progressive hepatic fibrosis, suggesting that Cldn5 downregulation may contribute to fibrotic remodeling during liver injury [150]. Although Cldn5 has received limited attention in in vitro liver studies, in vivo findings reveal sex- and age-dependent variation in its expression. For example, aged female mice (24 months old) exhibited higher hepatic Cldn5 levels than age-matched males, indicating a potential sex-specific regulation of Cldn5 with aging [151].

In the context of liver cancer, Cldn5 expression has been investigated in hepatocellular carcinoma (HCC). In a study analyzing 67 HCC specimens and 10 non-tumorous liver tissues, Cldn5 levels were significantly elevated in cancerous specimens compared to normal liver [150]. While the Human Protein Atlas confirms Cldn5 expression in HCC, its prognostic value for overall survival remains limited. Mechanistically, Cldn5 may contribute to endothelial responses during hepatic injury. In models of acute liver damage, oxidative stress and inflammation disrupt hepatocellular TJs, facilitating paracellular leakage across hepatic SECs, a pathological event associated with fibrosis and hepatocarcinogenesis [173]. In support of this, a recent study demonstrated that synthetic 4-phenyltetrahydroquinoline derivatives conferred hepatoprotection against carbon tetrachloride (CCl_4_)-induced liver injury. The protective effect was mediated by inhibition of oxidative stress and inflammation through autophagy suppression [174]. Although Cldn5 was not directly examined, such interventions may indirectly preserve TJ integrity, including that of Cldn5. While current evidence remains preliminary and direct mechanistic studies on Cldn5 are scarce, its elevated expression in aging, fibrotic, and malignant liver tissues suggests it may serve as a biomarker of hepatic endothelial health and a potential therapeutic target in liver disease.

### 7.4. Gut Health and Disease

The mucosal barrier of the gastrointestinal tract (GIT), formed by tight junctions, is essential for maintaining selective permeability and preventing the translocation of antigens from the lumen into the bloodstream [175]. Cldn5 exhibits a region-specific expression pattern and functional importance across the GIT. In murine models, Cldn5 is broadly expressed throughout the GIT, with prominent localization at the base of the intestinal crypts. In humans, Cldn5 is abundantly expressed in both the small intestine and colon [153]. Under physiological conditions, lamina propria lymphocytes upregulate Cldn5 to promote intestinal epithelial proliferation and differentiation via Notch-1 signaling, thereby contributing to epithelial homeostasis [154]. In vitro studies further support its functional significance. Cldn5 is detected in HT-29/B6 cells but absent in Caco-2 cells, both of which are human colorectal adenocarcinoma lines. Importantly, the transfection of Caco-2 cells with Cldn5 enhances epithelial barrier integrity, as evidenced by increased TEER and reduced paracellular permeability [176].

Research has shown that decreased levels of Cldn5 in several GI diseases compromise barrier integrity and increase intestinal permeability by disrupting tight junctions, thereby facilitating the passage of pro-inflammatory cytokines and promoting inflammation [177]. In both inflammatory bowel disease (IBD) patients and dextran sodium sulfate (DSS)-induced colitis models, elevated interleukin-21 (IL-21) levels are associated with Cldn5 downregulation, mediated by upregulation of miR-423-5p [153]. This microRNA, in turn, activates NF-κB/MAPK/JNK signaling pathways, ultimately compromising intestinal barrier function. In Crohn’s disease (CD), a specific subset of IBD, significant reductions in Cldn5 expression have been reported within the intestinal epithelium. During active disease phases, Cldn5 downregulation is associated with disrupted TJ architecture and impaired intestinal barrier integrity [155]. Remarkably, a study involving monozygotic twins discordant for CD revealed that even healthy co-twins exhibited lower Cldn5 levels compared to unrelated healthy controls, suggesting that genetic predisposition plays a role in Cldn5 regulation [156].

Additionally, vitamin D receptor (VDR) signaling has emerged as a key upstream regulator of Cldn5. Mice deficient in VDR show reduced Cldn5 mRNA expression, leading to increased intestinal permeability and elevated risk of colitis-associated tumorigenesis [157]. Conversely, epithelial-specific VDR overexpression restores Cldn5 levels and confers protection against inflammation-induced tumors, establishing Cldn5 as a downstream target of VDR and highlighting the VDR–Cldn5 axis as a potential therapeutic target in IBD [157].

Emerging evidence also links Cldn5 to modulation of the tumor–immune microenvironment. Bioinformatic analyses of colon adenocarcinoma datasets reveal that Cldn5 expression positively correlates with immune cell infiltration, including CD4+ and CD8+ T cells, macrophages, and dendritic cells [178]. This implies that Cldn5 may influence immune responses in addition to maintaining epithelial barrier function. Collectively, these findings underscore the multifaceted roles of Cldn5 in intestinal homeostasis, inflammation, and tumorigenesis. Future research should further elucidate its regulatory mechanisms and explore its potential as a therapeutic target in barrier-related gastrointestinal disorders.

### 7.5. Renal and Urinary Tract Barrier Function and Disease

Cldn5 plays a pivotal, though incompletely understood, role in maintaining the integrity of renal and urinary tract barriers. In the kidney, immature podocytes are initially connected by TJs, which are later remodeled into slit diaphragms as the intercellular spaces widen [179]. Cldn5 is abundantly expressed in the glomeruli, with podocytes being the exclusive cell type exhibiting this expression [158]. Cldn5 is also expressed in the glomerular capillary endothelial cells as well as renal arterial endothelium [27,180]. Although the role of Cldn5 in mature podocytes remains elusive, Cldn5 is localized to the plasma membrane of these cells under physiological conditions, suggesting a potential structural or signaling function [158]. To explore the role of Cldn5 involvement in diabetic kidney disease (DKD), its expression was assessed in two mouse models of diabetic nephropathy (DN): a unilateral nephrectomy combined with a streptozotocin (STZ)-induced type 1 diabetes model and the DB/DB type 2 diabetic mouse model. In both models, Cldn5 expression was significantly reduced in glomeruli, a finding corroborated by transcriptomic analyses of human kidney disease datasets [181]. To establish causality, a podocyte-specific Cldn5 knockout was generated in STZ-induced DN mice, revealing that loss of Cldn5 heightened susceptibility to diabetic kidney injury, as evidenced by early-onset albuminuria (at 4 weeks) and glomerulosclerosis (at 12 weeks) [159]. These data support a protective role for Cldn5 in podocyte stability and glomerular filtration barrier integrity. From a therapeutic standpoint, fluvoxamine, a known antidepressant with anti-inflammatory properties, attenuated LPS-induced acute renal injury by preserving Cldn5 and ZO-1 expression, highlighting a potential avenue for pharmacologic protection of renal TJs [162].

Interestingly, kidney dysfunction can also affect Cldn5 expression in distant organs. In a chronic kidney disease (CKD) mouse model, elevated systemic urea led to downregulation of Cldn5 in brain endothelial cells, with concomitant disruption of the BBB mediated by MMP2 activation [160]. Complementary findings have shown that CKD increases the risk of cerebral microbleeds by 2- to 2.5-fold, attributed to marked reductions in Cldn5 levels within brain microvessels, reinforcing the significance of cross-organ barrier interactions [161]. Moreover, aging has been linked to a significant decline in renal Cldn5 expression, suggesting that TJ integrity deteriorates with age, potentially contributing to renal epithelial barrier vulnerability in older individuals [151].

Shifting focus to the urinary bladder, Cldn5 also appears to contribute to urothelial barrier function. The urinary bladder serves as a critical component of the excretory system, efficiently storing urine and preventing leakage of its toxic and pathogenic contents into surrounding tissues or systemic circulation [182]. This barrier function is mediated primarily by the urothelium, a highly specialized, multilayered epithelium that provides frontline defense against urinary stones, toxins, and microbial invasion [183]. In situ hybridization studies have shown Cldn5 localization in the human ureteric urothelium, particularly at the basolateral junctions of superficial urothelial cells [184]. Data from the Human Protein Atlas indicates moderate to high Cldn5 expression in urothelial tissues, both in vivo and in vitro [185]. Despite this, direct evidence linking Cldn5 deficiency or dysfunction to urinary bladder pathologies remains limited. Future investigations are needed to elucidate how Cldn5 contributes to urothelial barrier permeability and whether it represents a viable therapeutic target in urinary tract disorders.

### 7.6. Skin Barrier Integrity and Dermatological Disorders

Cldn5 plays a critical role in maintaining the multifaceted barrier system in the skin, which is composed of TJs, the stratum corneum, resident microbiota, immune defenses, and chemical barriers [186]. This multilayered defense is essential for protecting the body against external insults and preventing transepidermal water and solute loss. In healthy interfollicular epidermis, TJ proteins such as occludin localize to the stratum granulosum, whereas Cldns and ZO-1 are distributed within the upper stratum spinosum [187]. In the skin, Cldn5 is primarily localized at the cell–cell borders of dermal vascular endothelial cells [188] and has also been observed in the granular cell layer of the epidermis [189]. In general, Cldn5 expression is largely endothelial, with only limited presence in keratinocytes of the epidermis. In mouse models with endothelial-specific deletion of Cldn5, there was a marked increase in 2MDa dextran leakage through ear skin microvessels, underscoring the importance of both epidermal and vascular Cldn5 in maintaining cutaneous barrier integrity [190].

Pathological skin conditions can markedly influence Cldn5 expression. In sensitive skin syndrome, where patients exhibit exaggerated responses to otherwise innocuous stimuli [164], Cldn5 knockdown in keratinocyte cultures and human organotypic skin models resulted in significantly reduced TEER and increased skin permeability. This phenotype was linked to overexpression of miRNA-224-5p, which likely contributes to permeability barrier disruption by suppressing Cldn5 levels [163]. In contrast, epidermodysplasia verruciformis (EpV), a genetic skin disorder associated with increased risk for cutaneous squamous cell carcinoma (cSCC), shows an opposite pattern [165]. Cldn5 expression was significantly elevated in malignant cSCC lesions compared to non-malignant flat warts, suggesting Cldn5 may serve as a prognostic biomarker for EpV-associated skin cancer progression. Interestingly, psoriatic lesions appear to maintain normal Cldn5 expression and localization. Studies report comparable Cldn5 levels in lesional and non-lesional epidermis, particularly in the granular layer [189]. Altogether, Cldn5 is a key TJ component supporting cutaneous vascular and epithelial barrier function, and dysregulation of its expression may be linked to altered vascular permeability, inflammation, and edema seen across various dermatologic conditions.

## 8. Targeting Cldn5 to Restore Barrier Integrity in Diseases

### 8.1. Therapeutic Modulation of Cldn5 to Enhance Drug Delivery

Interestingly, the therapeutic modulation of Cldn5 expression has been explored as a strategy to transiently and selectively open the iBRB, thereby facilitating drug delivery to the retina. This approach aims to overcome one of the primary challenges in treating retinal diseases, the restricted permeability of the iBRB to macromolecules and hydrophilic drugs. In a pivotal study, targeted delivery of siRNA against Cldn5 to retinal capillary endothelial cells in mice successfully induced a size-selective and reversible opening of the iBRB. This transient disruption permitted the passage of otherwise impermeable molecules, providing a promising platform for delivering therapeutics directly to the neural retina in degenerative retinal diseases [191].

A subsequent investigation further validated the temporal and reversible nature of this strategy. Systemic administration of Cldn5 siRNA led to maximal suppression of Cldn5 expression approximately 48 h post-injection. During this window, both the iBRB and the BBB exhibited increased permeability to Hoechst dye (563 Da), confirming that systemic modulation of Cldn5 enables controlled and transient barrier opening [192]. Together, these findings suggest that regulated suppression of Cldn5 can be harnessed to enhance drug delivery across the iBRB, potentially transforming the treatment landscape for retinal degenerative conditions. However, further studies are needed to evaluate the safety, specificity, and clinical applicability of this approach.

### 8.2. Modulation of Cldn5 Expression as a Therapeutic Strategy in DR

Disruption of the BRB is a defining feature of ischemic retinopathies. Therefore, targeting Cldn5 expression and localization as an integral TJ protein that maintains endothelial cell–cell adhesion and controls paracellular permeability at the BRB offers the potential to both restore barrier function and control pathological neovascularization (Table 2).

While transient suppression of Cldn5 can be therapeutically advantageous for enhancing retinal drug delivery, sustained downregulation of Cldn5 is a pathological feature associated with diabetic retinal barrier breakdown. In this context, therapeutic approaches aimed at restoring Cldn5 expression or promoting its proper junctional localization have demonstrated significant potential for preserving or re-establishing BRB integrity. One such strategy involves the use of Norrin, a secreted ligand critical for retinal vascular development and barrier formation. Norrin has been shown to effectively counteract VEGF-induced hyperpermeability in both in vitro and in vivo models and to ameliorate BRB dysfunction in the context of diabetes [193,194]. Mechanistically, Norrin acts through the Frizzled-4 (FZD4) receptor and its downstream effector Disheveled-1 (DVL-1), a scaffold protein that co-localizes with and directly binds to Cldn5. This interaction supports the stabilization and retention of Cldn5 at endothelial junctions, thereby enhancing barrier function and mitigating pathological leakage [195]. These findings position Norrin signaling as a promising therapeutic axis for reestablishing Cldn5-mediated TJ integrity and combating retinal vascular permeability in DR.

In addition to genetic and ligand-based strategies, pharmacological agents have shown promise in preserving or restoring Cldn5-mediated barrier function in DR. Several compounds act by stabilizing TJ architecture and counteracting the deleterious effects of hyperglycemia and inflammation on the BRB. FTY720 (Fingolimod), an immunomodulatory drug that targets sphingosine-1-phosphate receptors (S1PRs), has been shown to protect against diabetes-induced retinal barrier disruption [199]. In STZ-induced diabetic rats, FTY720 preserved the expression of key TJ proteins, including Cldn5, ZO-1, and occludin, thereby maintaining barrier integrity [199]. In ROP, where Cldn5 expression is upregulated, agents such as siRNAs or triciribine [43] may be utilized as potential therapeutics.

Another molecular target implicated in TJ regulation is basigin, a membrane-bound protein involved in cytokine-induced endothelial dysfunction. In diabetic retinas, basigin mediates the mislocalization of Cldn5, contributing to barrier breakdown [196]. Notably, silencing basigin using siRNA in endothelial cells prevented VEGF- and TNF-α–induced suppression of Cldn5, restoring its proper localization at endothelial junctions. This effect was confirmed in diabetic mouse retinal flat mounts, supporting basigin as a regulator of junctional integrity under pathological conditions [196,197].

Together, these findings highlight the potential of pharmacological interventions targeting signaling pathways and membrane regulators to reinforce Cldn5 expression and localization, offering viable strategies to preserve BRB function in DR. However, the in vitro elevation of Cldn5 expression in HRECs following HG and AGE exposure, along with the increased vitreous levels of circulating Cldn5 in DR patients, warrants further investigation before pursuing therapeutic modulation of retinal Cldn5 in DR.

### 8.3. Potential Utility of Cldn5 as a Functional Reporter and a Screening Target

Recent technological advances have enabled dynamic, real-time visualization of Cldn5 expression through the development of a Cldn5-GFP reporter system. This genetically engineered model emits green fluorescence in patterns that correspond to TJ localization and endothelial barrier integrity, providing a valuable tool for assessing BRB status under both physiological and pathological conditions [198]. Using this reporter model, the tyrosine kinase inhibitor RepSox was identified as a potent inducer of Cldn5 expression. Treatment with RepSox significantly enhanced endothelial resistance, reinforcing TJ structure and function [198]. These results suggest that RepSox or similar compounds could have therapeutic potential for DR and related ischemic retinal disorders by strengthening barrier integrity. Collectively, these findings reinforce the dual utility of Cldn5 as both a dynamic biomarker for retinal vascular health and a modulatable therapeutic target in ischemic retinopathies.

### 8.4. Targeting Cldn5 to Restore Barrier Integrity in Cancers and Other Diseases

Cldn5 downregulation is a common feature in several tumor types, such as breast invasive carcinoma, cervical cancer, and lung adenocarcinomas and squamous cell carcinomas, where it contributes to the compromise of the blood–tumor barrier, thereby facilitating vascular leakage, tumor cell infiltration, and metastasis [42]. Conversely, enhancing Cldn5 expression can reinforce barrier integrity and suppress tumor invasion. For instance, overexpression of Cldn5 in human brain endothelial cells significantly reduced A549 lung adenocarcinoma cell transmigration across an endothelial monolayer in vitro [65]. Mechanistically, tumor progression often involves epithelial–mesenchymal transition (EMT), during which epithelial markers like Cldn5 are downregulated [42]. Notably, in ovarian cancer, SIRT1 activation promotes deacetylation and nuclear translocation of KLF4, a transcriptional activator of Cldn5, thereby enhancing its expression, suppressing EMT, and reducing migratory capacity [200]. Together, these findings indicate the pivotal role of Cldn5 in maintaining endothelial barrier integrity and highlight its therapeutic potential in limiting tumor progression and metastasis.

Several compounds have also been shown to stabilize Cldn5 at the blood–brain barrier by targeting upstream regulatory mechanisms. Valproic acid and GSK-3β inhibitors elevate β-catenin levels [201], supporting adherens and tight junction stability and prolonging Cldn5 half-life [112,202]. Inhibition of TGF-β signaling with agents like Sunitinib can also restore barrier integrity by preventing tyrosine phosphorylation–mediated disruption of VE-cadherin and Cldn5 [112,127]. The broader therapeutic potential of Cldn5 modulation in other disease contexts remains largely unexplored and warrants further investigation.

## 9. Conclusions and Future Directions

The comprehensive review of Cldn5 across various vascular beds highlights its pivotal and multifaceted role in maintaining blood–tissue barrier integrity, with its dysregulation being a common feature across a wide spectrum of physiological and pathological conditions, from neuroinflammation and cardiovascular injury to organ-specific barrier dysfunction. The primary strength of the current research is the extensive evidence demonstrating the critical role of Cldn5 in a diverse range of diseases, particularly in the BBB and BRB, where its downregulation is a clear hallmark of barrier compromise. This has led to promising therapeutic strategies like RNA silencing and pharmacological stabilizers in preclinical models, further supported by its potential as a dual-role protective factor and a biomarker, with altered serum levels correlating to various psychiatric and inflammatory conditions.

However, several significant challenges and weaknesses persist in the field. The most notable is the paradoxical nature of Cldn5 expression, where both reduced and excessive levels can lead to barrier dysfunction, particularly in the lung, brain, and retina, with other organs underexplored. This context-dependent behavior, exemplified by its overexpression paradoxically impairing barrier resistance in DR, necessitates a more nuanced approach to therapeutic intervention. Compounding this issue are the “discrepancies in the literature” regarding its expression and function, including conflicting results from human studies on conditions like schizophrenia, which suggests its role may be influenced by temporal factors, demographics, or specific disease subsets. Furthermore, there is a scarcity of direct mechanistic studies in less-studied organs like the liver and gut, where their involvement in fibrotic remodeling and IBD is suggested but not yet fully elucidated. The recent discovery of extracellular vesicle-mediated Cldn5 transfer also presents a complex, novel mechanism that challenges traditional understanding and warrants further investigation.

A major challenge in interpreting the role of Cldn5 in health and disease is disentangling whether observed changes in expression are causally driving pathology or simply reflective of broader cellular stress or remodeling. In many studies, altered Cldn5 levels correlate with disrupted barrier function or disease progression, but definitive proof of causality, particularly in human tissues, remains limited. Furthermore, the consequences of Cldn5 dysregulation are highly context-dependent: in some conditions, downregulation compromises the endothelial barrier and promotes inflammation or leakage, while in others, upregulation of Cldn5 has been linked to pathological stiffening or impaired cell turnover. This duality is evident in several settings, including aging and senescence, where both increases and decreases in Cldn5 expression have been associated with deleterious outcomes depending on the tissue type, disease stage, or microenvironmental cues. As highlighted in Figure 4, this complexity signifies the need for careful interpretation of Cldn5 changes and supports the importance of context-specific models in understanding its role in disease mechanisms.

The observation that both upregulation and downregulation of Cldn5 can be associated with pathological outcomes likely reflects the complex, context-dependent roles of this protein across different tissues and disease states. In some settings, such as the BBB, reduced Cldn5 expression clearly compromises endothelial integrity and promotes inflammation. However, in other contexts, such as retinal inflammation, tumor microenvironments, or aging, excessive Cldn5 expression may contribute to pathology by excessively tightening barriers, limiting immune surveillance, or altering endothelial plasticity. These paradoxical effects may also be explained by temporal dynamics, where the impact of Cldn5 changes varies across disease progression, or by compensatory responses, where altered expression reflects an adaptive, yet potentially maladaptive, reaction to cellular stress or damage. Moreover, Cldn5 may exert functions beyond barrier regulation, including effects on cytoskeletal organization and intracellular signaling, which could drive divergent outcomes depending on the surrounding molecular landscape [56]. Finally, because TJs function as multi-protein complexes, the consequences of Cldn5 dysregulation may depend on the status of interacting proteins such as ZO-1 or occludin, further contributing to the variability in observed effects.

In order to harness the full potential of Cldn5 as a therapeutic target, future research must focus on resolving these existing discrepancies and understanding its context-specific functions. This includes detailed mechanistic studies to clarify its dual role as both protective and harmful, and the development of organ- and disease-specific therapies to avoid unintended side effects. Additionally, further investigation into novel regulatory pathways, such as EVs, is essential to fully grasp the dynamic function of Cldn5. Future studies should aim to decipher the specific cargo and signaling molecules carried by these Cldn5+ EVs, as well as their precise role in intercellular communication and disease pathogenesis across organs. A concerted effort to standardize methodologies for measuring circulating Cldn5 is also necessary to establish its reliable use as a clinical biomarker. By addressing these challenges, researchers can effectively develop targeted therapies for a multitude of diseases characterized by compromised vascular barrier function.

## Figures and Tables

**Table 1:** A summary of Cldn5 expression, function, and regulation by tissue.

Organ	Cell Type	Regulation	Mechanisms/Physiology/Pathology	Consequences	Ref.
Brain (BBB)	BMECs	Down	Aging (early event) Amyloid-laden microvessels (Cerebral amyloid angiopathy) Mild cognitive impairment Alzheimer’s Disease Epilepsy Traumatic Brain Injury Multiple Sclerosis Inflammatory cytokines (IL-1β) HIV-1 (Tat protein) HG and AGE (via EV shedding) CCL2 (caveolin-dependent endocytosis) Hypoxia/Ischemia (cytosolic accumulation).	Size-selective BBB loosening precedes cognitive impairment and cerebral hemorrhage, facilitating neuroinflammation and immune cell infiltration into the CNS, exacerbating barrier breakdown through TJ disassembly and fragmented Cldn5 immunoreactivity, particularly in conditions like HIV.	[51,59,61,62,63,71,74,87,88,89]
		Up	Selective insulin receptor (IR) activation (restores AKT2 signaling) Glial-neuronal interactions *Cryptococcus neoformans* infection Mesenchymal stem cell–derived EVs (prevent Caveolin-1 mediated endocytosis).	Restore BBB integrity by reinforcing TJs, promote EC proliferation, migration, and adhesion—mechanisms that impede cancer metastasis.	[53,63,65,72,73,75,76]
Retina (iBRB)	RECs; Retinal Pigment Epithelium (RPE)	Down	HG MMP-2, MMP-9 Hypoxia VEGF/ROCK1 axis (destabilization).	Increased paracellular permeability, reduced TEER, increased retinal capillary permeability, and impaired barrier function	[103,107,109,117]
		Up; Mis- localization	HG + AGE Osteopontin OIR	Impaired HREC barrier function, often due to mislocalization to cytosolic/ non-junctional regions), promoting pathological angiogenesis (OIR).	[43,45,106,120,122]
Peripheral Nerves (BNB)	Endoneurial ECs	Down	Guillain–Barré syndrome; Chronic inflammatory demyelinating polyneuropathy	Compromises BNB, allowing immune cells and antibodies to infiltrate and attack peripheral nerve myelin.	[131]
		Up	Corticosteroids (hydrocortisone) Upregulated by GDNF released by pericytes.	Tightens BNB, reduces permeability, restorative effect in neuropathies, helps maintain barrier integrity in nerves.	[132,133]
Cardiovascular System	Cardiomyocytes, ECs	Down	Dystrophin-utrophin double knockout (*Dmdmdx;Utrn^−/−^ dko*) mouse model Acute myocardial ischemia/IR injury Hypoxia and reoxygenation in cardiomyocytes Atrial fibrillation (left atrial appendage) Ventricular dysfunction, Heart Failure.	Contributes to cardiac injury, cardiac dysfunction associated with dilated cardiomyopathy (advanced stages), structural abnormalities and heart failure.	[134,135,136,137,138,139,140]
		Up	Cardiac-specific Cldn5 overexpression (adenovirus).	Mitigates oxidative stress, inflammation, apoptosis, and cardiac dysfunction. Cardioprotective (inhibits cardiomyopathy hallmarks) and acts as a histological indicator of cardiac injury.	[136,137]
Lung (Alveolar-Capillary Barrier	Microvasculature; Alveolar Epithelium	Down	Hazardous gas (hydrogen sulfide) HIV-1 infection Neuroinflammation due to severe bacterial lung infections Lung squamous cell carcinoma	Induces acute lung injury, contributes to interstitial pneumonitis and vascular injury, perturbs the BBB (secondary to lung infection), and suppresses tumors upon overexpression.	[141,142,143,144,145]
		Up	Simvastatin (enhances endothelial barrier via VE-cadherin–dependent pathway involving FoxO1 and β-catenin) AGE (in epithelial/endothelial cells) (detrimental).	Enhances the endothelial barrier and paracellular barrier function. AGE disrupts epithelial barrier function and significantly reduces TEER.	[146,147,148,149]
Liver	Sinusoidal ECs; Arterial/Portal Veins	Down	Progressive hepatic fibrosis.	Correlated with hepatic injury, fibrotic remodeling.	[150]
		Up	Aging (in old female mice) Hepatocellular carcinoma (HCC).	Biomarker of hepatic vascular health and HCC progression.	[151,152]
Gut	Intestinal Epithelial Cells, ECs	Up	Lamina propria lymphocytes (promotes via Notch-1 signaling).	Promotes intestinal epithelium proliferation, differentiation, and maintains epithelial homeostasis.	[153,154]
		Down	Crohn’s disease Genetic predisposition (monozygotic twins discordant)	Compromises intestinal barrier integrity, disrupts TJ architecture, increases intestinal permeability, and higher incidence of colitis-associated tumors.	[153,155,156,157]
Kidney	Podocytes and glomerular capillaries	Down	In Diabetes, DKD, CKD, and aging	Causes albuminuria, glomerulosclerosis, increased microhemorrhages, and barrier fragility.	[151,158,159,160,161]
		Up	With Fluvoxamine treatment in kidney injury	Protection from LPS-induced kidney injury.	[162]
Skin	Superficial layers, ECs	Down	In sensitive skin Endothelial-specific KO	Vascular leakage, increased permeability, and reduced TEER in sensitive skin.	[163,164]
		Up	In cSCC	A marker of tumor progression.	[165]

**Table 2:** Pharmacological and genetic modulation of Cldn5: Mechanisms and barrier function outcomes across organ systems.

Organ	Interventions	Approach/Physiology/Pathology	Consequences	References
Brain	Therapeutic modulation	Low intravenous anti-Cldn5 monoclonal antibody, M01 (Cldn5 inhibitor).	Increased CSF tracer levels (improved permeability) Transient barrier opening (blood-spinal cord barrier) Decreased neuroinflammation Reduced vasogenic edema (spinal cord injury).	[90,92]
	Restoration	Intravenous administration of 4 μM Cldn5.	Restored hippocampal Cldn5 mRNA levels and improved learning and memory.	[84]
Retina	Restoration	Norrin signaling (stabilizes TJs via Disheveled-1 binding) ROCK1 inhibitors (restabilizes distribution, boosts transcription) Silencing Basigin (prevents VEGF/TNF-α induced suppression) RepSox (tyrosine kinase inhibitor, identified via reporter system), autophagy (preserves and maintains membrane localization under hypoxia/ischemia).	Reverses VEGF-induced hyperpermeability Mitigates diabetes-associated barrier disruption Restores TJ localization Enhances endothelial resistance and restores TEER values.	[74,193,194,195,196,197,198]
	Therapeutic modulation	siRNA against Cldn5 (targeted delivery). Triciribine treatment in OIR mice retinas	Induces size-selective, reversible opening of iBRB, improves drug delivery, and provides a temporal control of barrier permeability. Reduces neovascular tuft formation and vascular leakage	[43,191,192]
Gut	Restoration	Epithelial-specific Vitamin D receptor (VDR) overexpression.	Restores Cldn5 levels, protects against inflammation-induced tumorigenesis, and highlights VDR-Cldn5 axis as therapeutic target in IBD.	[157]

## Data Availability

There were no new data created in this study.

## Abbreviations

The following abbreviations are used in this manuscript:

BBB	Blood–Brain Barrier
BRB	Blood–Retinal Barrier
iBRB	Inner Blood–Retinal Barrier
oBRB	Outer Blood–Retinal Barrier
Cldn5	Claudin-5
TJs	Tight Junctions
ECs	Endothelial Cells
EVs	Extracellular Vesicles
CNS	Central Nervous System
ZO-1	Zonula Occludens-1
GDNF	Glial Cell Line-Derived Neurotrophic Factor
MAPK	Mitogen-Activated Protein Kinase
ERK	Extracellular Signal-Regulated Kinase
PI3K	Phosphoinositide 3-Kinase
Akt	Protein Kinase B
FOXO1	Forkhead Box O1
MS	Multiple Sclerosis
EAE	Experimental Autoimmune Encephalomyelitis
CD	Cluster of Differentiation (e.g., CD4^+^ T cells)
HG	High Glucose
AGE	Advanced Glycation End Products
TEER	Transendothelial/Transepithelial Electrical Resistance
HIV	Human Immunodeficiency Virus
OCD	Obsessive–Compulsive Disorder
IL-6	Interleukin-6
IR	Insulin Receptor
VEC	Vascular Endothelial Cadherin
VDR	Vitamin D Receptor
IBD	Inflammatory Bowel Disease
PBS	Phosphate-Buffered Saline
DKD	Diabetic Kidney Disease
DN	Diabetic Nephropathy
CKD	Chronic Kidney Disease
cSCC	Cutaneous Squamous Cell Carcinoma
EpV	Epidermodysplasia Verruciformis
LPS	Lipopolysaccharide
MMP3	Matrix Metalloproteinase 3
JAM-A	Junctional Adhesion Molecule-A
VE-cadherin	Vascular Endothelial Cadherin
SH-SY5Y	Human Neuroblastoma Cell Line
U251	Human Glioblastoma Astrocytoma Cell Line
hCMEC/D3	Human Cerebral Microvascular Endothelial Cells (immortalized line)
